# A comparative glycoproteome study of developing endosperm in the hexose-deficient *miniature*1 (*mn*1) seed mutant and its wild type *Mn*1 in maize

**DOI:** 10.3389/fpls.2014.00063

**Published:** 2014-02-26

**Authors:** Cecilia Silva-Sanchez, Sixue Chen, Jinxi Li, Prem S. Chourey

**Affiliations:** ^1^Proteomics, Interdisciplinary Center for Biotechnology Research, University of FloridaGainesville, FL, USA; ^2^Department of Biology, UF Genetics Institute, University of FloridaGainesville, FL, USA; ^3^United States Department of Agriculture, Agricultural Research Service, Center for Medical, Agricultural and Veterinary EntomologyGainesville, FL, USA; ^4^Department of Agronomy, University of FloridaGainesville, FL, USA

**Keywords:** seed development, gene expression, sugar methabolism, transfer cells, maize

## Abstract

In maize developing seeds, transfer cells are prominently located at the basal endosperm transfer layer (BETL). As the first filial cell layer, BETL is a gateway to sugars, nutrients and water from mother plant; and anchor of numerous functions such as sucrose turnover, auxin and cytokinin biosynthesis/accumulation, energy metabolism, defense response, and signaling between maternal and filial generations. Previous studies showed that basal developing endosperms of *miniature*1 (*mn*1) mutant seeds lacking the *Mn*1-encoded cell wall invertase II, are also deficient for hexose. Given the role of glucose as one of the key sugars in protein glycosylation and proper protein folding; we performed a comparative large scale glycoproteome profiling of total proteins of these two genotypes (*mn*1 mutant vs. *Mn*1 wild type) using 2D gel electrophoresis and glycosylation/total protein staining, followed by image analysis. Protein identification was done by LC-MS/MS. A total of 413 spots were detected; from which, 113 spots matched between the two genotypes. Of these, 45 showed >20% decrease/increase in glycosylation level and were selected for protein identification. A large number of identified proteins showed decreased glycosylation levels in *mn*1 developing endosperms as compared to the *Mn*1. Functional classification of proteins, showed mainly of post-translational modification, protein turnover, chaperone activities, carbohydrate and amino acid biosynthesis/transport, and cell wall biosynthesis. These proteins and activities were related to endoplasmic reticulum (ER) stress and unfolded protein response (UPR) as a result of the low glycolsylation levels of the mutant proteins. Overall, these results provide for the first time a global glycoproteome profile of maize BETL-enriched basal endosperm to better understand their role in seed development in maize.

## Introduction

The *mn*1 mutation in maize is associated with a loss of 70% seed weight at maturity due to a loss-of-function mutation at the *Mn*1 locus that codes for the endosperm-specific cell wall invertase 2 (INCW2). The INCW2 protein is entirely localized in basal endosperm transfer cell layer (BETL), a major if not the sole gateway for the intake of sugars and nutrients from maternal cells into the filial tissue (Miller and Chourey, 1992; Cheng et al., 1996). Homozygous *mn*1 mutant is not lethal presumably due to a residual low level of the cell wall invertase (CWI) activity encoded by another homolog, *Incw*1 (Chourey et al., 2006). A BETL in developing maize seed is composed of two to three strata of highly specialized transfer cells (Davis et al., 1990; Kang et al., 2009), marked by a hallmark feature of labyrinth-like proliferation of cell wall called wall-in-growths (WIGs) in plants (Offler et al., 2003; Vaughn et al., 2007; Ruan et al., 2010). The INCW2 protein is immunolocalized to the *Mn*1 WIGs, and the *mn*1 mutant is associated with stunted WIG development due to the INCW2 deficiency (Kang et al., 2009). Sucrose hydrolysis is clearly a major physiological function of the *Mn*1-encoded INCW2 in the BETL and there is evidence that metabolically released hexoses, not exogenous, are critical as a driving force in assimilate movement between maternal and filial cells (Cheng and Chourey, 1999). As expected from the INCW2-deficiency, the *mn*1 basal endosperm shows greatly reduced levels of glucose, fructose and sorbitol; and increased levels of sucrose relative to the *Mn*1 developing seeds (LeClere et al., 2010; Chourey et al., 2012).

Glycoproteins (GPs) have one or more covalently attached glycan (oligosaccharide, often with the presence of glucose) moieties. Nearly all *N*-linked GPs are believed to be secretory proteins, which are associated with numerous functions, including stress tolerance and cell-cell communication in development. Proteins entering the secretory pathway are marked by signal peptides; 17% of all proteins in Arabidopsis have predicted signal peptides and, 33% of these have at least one trans-membrane domain associated with the endoplasmic reticulum (ER). Addition of *N*-glycans to the proteins occurs as a co-translational modification at the ER and then transported through secretory pathway to the cell surface in a traffic process mediated mostly by the Golgi complex (Vitale and Boston, 2008; Liu and Howell, 2010). The ER is suggested to be a nursery where newly synthesized secretory proteins are properly decorated, modified, de-decorated (deglycosylated), finally properly folded and assembled prior to exit to their final destination (Vitale and Boston, 2008). Quality Control (QC) in this entire process is highly critical. Improperly folded proteins in the ER are causal to the so called ER-stress that is associated with unfolded protein response (UPR)—a signal transduction pathway that is well studied in mammalian and yeast cells. Recent studies in plants suggest that QC, ER stress, and UPR play an important role in biotic/abiotic stress and seed development (Liu and Howell, 2010).

The most detailed studies of GPs in maize developing endosperm have been on the storage protein zein, stored in ER-derived protein bodies (Vitale and Boston, 2008; Arcalis et al., 2010). However, there is no such information on the GPs in the BETL, a cell type not associated with storage functions in seed development. Here we report the first detailed profile of the GPs in the basal endosperm using LC-MS/MS approach, a preeminent tool for identification and quantitative characterization of GPs (Ruiz-May et al., 2012). Comparative GP profiles of the BETL enriched proteins of the *mn*1 relative to the *Mn*1 revealed potential changes due to both the hexose deficiency.

## Experimental procedures

### Plant materials and chemicals

Immature maize (*Zea mays* L.) kernels wild type (*Mn*1) and *miniature 1* mutant (*mn*1) in the W22 inbred line were harvested at 12 days after pollination (DAP). All plants were grown in the field and were self- or -sib-pollinated. At the time of harvest, kernels were individually excised from the ear with a paring knife, taking care to include undamaged base (pedicel) of each kernel. Excised kernels were flash frozen in liquid nitrogen and stored at −80°C until analysis. We used the basal 1/3 end of the endosperm because it is enriched for the BETL cells, the sole site of the *Mn*1 expression and also a major zone for sucrose turn-over reactions, as previously discussed (LeClere et al., 2008; Silva-Sanchez et al., 2013). All chemicals were purchased from Fisher Scientific Inc., USA unless otherwise stated.

### Total protein extraction

Soluble proteins were extracted from the one-third lower part of maize kernels according to a method reported by Hurkman and Tanaka (1986) with minor modifications. The frozen kernels were ground into fine powder in a pre-chilled mortar and pestle. A total of 3 mL of extraction buffer (0.1 M Tris HCl pH 8.8, 10 mM EDTA, 0.2 M DTT, 0.9 M sucrose) and 3 mL of saturated phenol were added to homogenize the sample. To extract proteins, samples were constantly agitated for 2 h at room temperature. The mixture was then centrifuged at 5000 × g for 10 min at 4°C. The top clear phenol phase was removed and collected in a fresh tube. The remaining pellet was re-extracted with 3 mL buffered phenol solution and centrifuged again to recover the top clear phenol phase and combined with the previous fraction. The supernatant was precipitated with five volumes of ice cold 0.1 M ammonium acetate in 100% methanol overnight and centrifuged at 20000 × g for 20 min at 4°C. The supernatant was discarded and the pellet was washed twice with 0.1 M ammonium acetate in 100% methanol, then washed twice with cold 80% acetone and finally with 70% of ethanol. The pellet was solubilized in isoelectric focusing (IEF) buffer (7 M urea, 2 M thiourea, 4% (w/v) CHAPS and 40 mM DTT). The samples were treated with benzonase (Novagen, Gibbstown, NJ) for 30 min and then centrifuged at 34,000 rpm for 30 min at 15°C using an ultracentrifuge (Optima TLX ultracentrifuge, Beckman Coulter). Supernatant was collected and 50 μ L aliquots were kept at −80°C until use.

### Two dimensional gel electrophoresis, protein staining and imaging analysis

Protein samples were quantified using an EZQ protein quantitation kit (Invitrogen, CA, USA). Protein extracts of 500 μ g each were mixed with 0.2% of ampholytes (pH 3–10) and loaded onto an 18 cm Immobilized pH gradient (IPG) strips (pH range 3–10 NL) (GE healthcare, CA, USA), followed by overnight rehydration at room temperature. IEF was conducted on an Ettan IPGphor3 system (GE Healthcare, CA, USA) using the following conditions: 200 V for 30 min, then ramping to 500 V for 30 min, and finally to 10,000 V for 1 h. The voltage was held at 10,000 V until 85,000 Vh were reached in order to ensure complete separation and focusing of the proteins. After IEF, the strips were equilibrated for 15 min in equilibration buffer (6 M urea, 75 mM Tris-HCl pH 8.8, 29.3% glycerol, 2% SDS, 0.002% bromophenol blue) containing 2% DTT, and for another 15 min in equilibration buffer containing 2.5% iodoacetamide. The IPG strips were placed onto 18 cm 12.5% SDS gels (Jule Biotechnologies INC, Milford, CT, USA). Electrophoresis was run at 15 W for 5 h. A total of three replicate 2-DE experiments were conducted for each sample.

After gel electrophoresis, staining with a glycoprotein-specific stain Pro-Q Emerald fluorescent dye was performed according to manufacturer's instructions (Invitrogen, CA, USA). Images were acquired using the Investigator ProPic unit equipped with a UV light-box (Genomics solutions, MI, USA). Then, the gels were destained and restained with a total protein dye Sypro Ruby following manufacturer's instructions (Invitrogen, CA, USA). Images were acquired with the Investigator ProPic unit. Spot detection, matching and quantification across the replicate gels of wild type and mutant samples, were done using a Progenesis Samespot Software (Non-linear Dynamics, CA, USA). The function of automatic spot detection and matching was used, followed by manual inspection. Protein experimental molecular weights were calculated using a CandyCane™ molecular mass standard (Invitrogen, CA, USA) separated in a separate lane on the 2D gels, and the experimental isoelectric points were determined based on IPG strip specifications. Normalized spot volumes were used to determine the quantitative changes of glycosylated/total protein ratios and total protein levels. ANOVA test was used to determine the statistical significance.

### Protein identification using liquid chromatography tandem MS (LC-MS/MS)

Selected 2D gel spots were excised using an Investigator ProPic robot (Genomics Solutions Inc., USA) and digested with trypsin as previously described (Sheffield et al., 2006); The lyophilized peptides were resuspended in 15 μ L of loading buffer (3% acetonitrile, 0.1% acetic acid, 0.01% trifluoroacetic acid) and loaded onto a C18 capillary trap cartridge (LC Packings, USA) and then separated on a 15 cm nanoflow analytical C18 column (PepMap 75 μm id, 3 μm, 100 Å) at a flow rate of 300 nL/min on a nanoLC ultra 1D plus system (ABsciex, USA). Solvent A composition was 3% acetonitrile (ACN) v/v, 0.1% acetic acid v/v; whereas solvent B was 97% ACN v/v, 0.1% acetic acid v/v. Peptide separation was performed with a linear gradient from 3 to 40% of solvent B for 20 min, followed by an increasing to 90% of solvent B in 5 min and hold for 5 min (Zhu et al., 2010). The eluted peptides were directly sprayed into an LTQ Orbitrap XL mass spectrometer (Thermo Scientific Inc., Bremen, Germany). MS2 spectra were acquired in a data-dependent mode. An Orbitrap full MS scan (resolution: 3 × 10^4^, mass range 400–1800 Da) was followed by 10 MS2 scans in the ion trap, which were performed via collision induced dissociation on the top 10 most abundant ions. Isolation window for ion selection was 3 Da. Normalized collision energy was set at 28%. Dynamic exclusion time was 20 s (Li et al., 2012a). The acquired mass spectra were searched against a Uniprot *Zea mays* database (62,860 entries, Jan-04-2013) using Mascot 2.2 search engine (http://www.matrixscience.com) with the following parameters: tryptic peptides with 1 missed cleavage site, mass tolerance of precursor ion of 10 ppm and MS/MS ion of 0.8 Da, fixed carbamidomethylation of cysteine, variable methionine oxidation, asparagine and glutamine deamination. Unambiguous identification was done using Scaffold software V 3.0 (Proteome Software, OR, USA).

### Bioinformatics analysis

Functional classification of the proteins was done according to the clusters of orthologous groups for eukaryotic complete genomes (KOG) (ftp://ftp.ncbi.nih.gov/pub/COG/KOG/) (Tatusov et al., 2003) and the prediction of subcellular localization was done using a Plant-mPLoc tool (http://www.csbio.sjtu.edu.cn/bioinf/plant-multi/) (Chou and Shen, 2010).The identified proteins were submitted to an *N*-Glycosite tool for glycosylation site analysis (http://www.hiv.lanl.gov/content/sequence/GLYCOSITE/glycosite.html) (Zhang et al., 2004). Prediction of the N-terminal presequences: chloroplast transit peptide (cTP), mitochondrial targeting peptide (mTP) and secretory pathway signal peptide (SP) was done using the TargetP 1.1 tool (http://www.cbs.dtu.dk/services/TargetP/) (Emanuelsson et al., 2000).

## Results and discussion

### Two dimensional gel analysis, protein identification and glycoprotein (GP) detection

**Figure 1:**
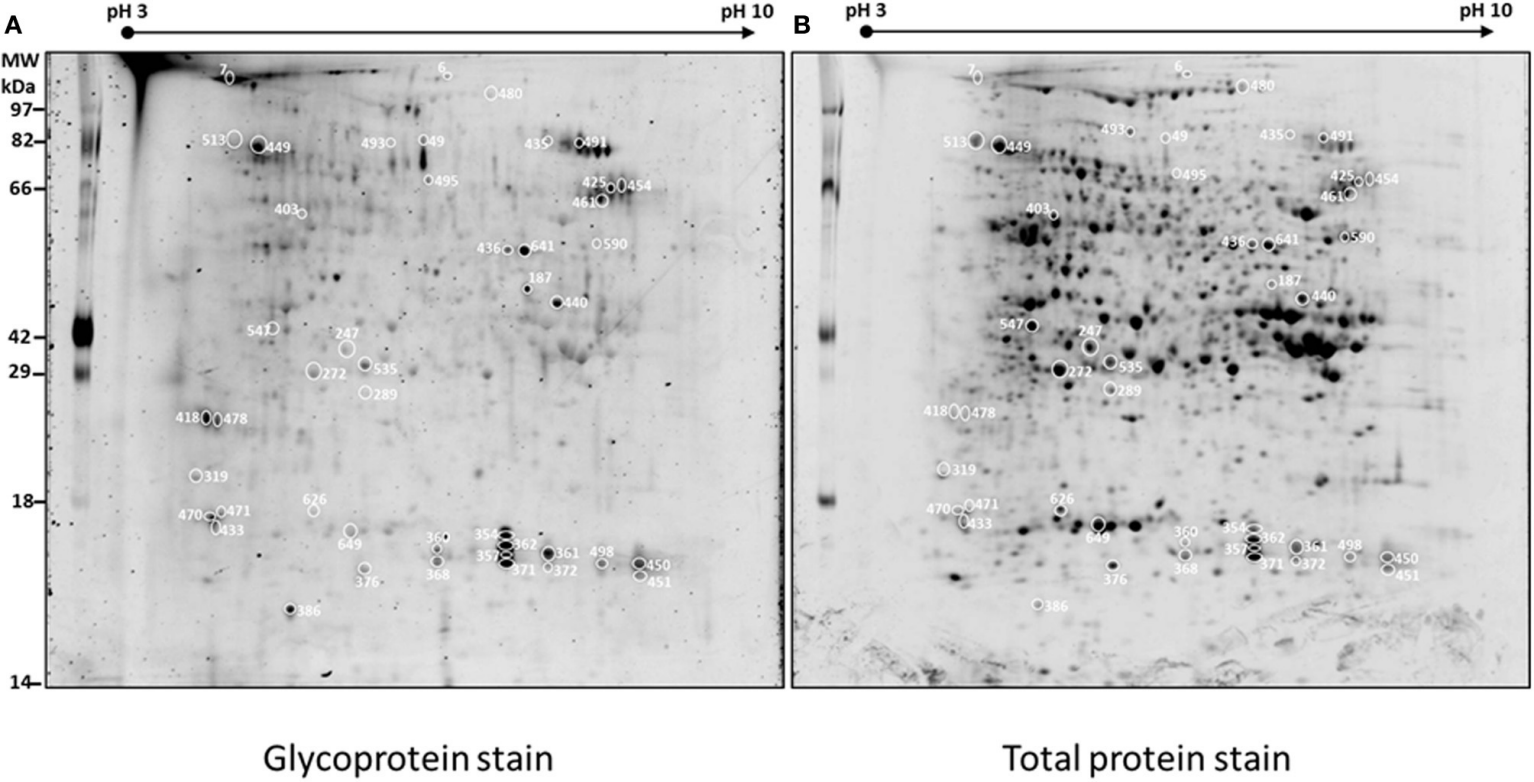
**Representative 2D-PAGE of BETL total protein extract stained with ProQ emerald for glycoprotein detection (A) and Sypro ruby for total protein detection (B)**. The spots along with the spot numbers selected for protein identification by LC-MS/MS are marked with a circle. Numbers on various spots in each gel refer to the #s shown in the 1st column, Table 1.

In order to determine the differences in glycosylation patterns of BETL between the wild type (WT) and the mutant (M), total protein extracts were prepared from the one-third lower part of maize kernels. For each sample, three biological replicates/gels were performed. The gels were stained with ProQ Emerald for glycoproteins, and then for total protein detection with Sypro Ruby stain. Figure 1 shows representative images of gels stained for potential glycoproteins and total proteins. A total of 413 spots were detected in the four gels of the three replicates (Supplemental Figure 1) representing the wild type and mutant endosperm extracts; of these, 254 spots with *p*-values smaller than 0.05 based on ANOVA test were selected for further analysis.

Glycosylation of each protein spot was evaluated by their staining intensity with the GP stain that was normalized by the intensity of total protein stain (G/TP) for both WT and the mutant samples. Normalized glycosylation ratios are represented as *M*_(*G*/*TP*)_/*WT*_(*G*/*TP*)_ in Table 1. A total of 113 protein spots matched between the wild type and mutant gels, are shown in Supplemental Table 1. There are 45 spots (Figure 1) showing more than 20% increase or decrease in the normalized glycosylation level. Interestingly, only 4% of the 113 spots matched between WT and M showed increased GP ratios when comparing to the WT, while 36% showed decreased ratios (Supplemental Table 1).

**Table 1:** **Glycoproteins identified in wild type (*Mn1*) and mutant *mn1-1* BETL protein extracts**.

**#**	**Accession number**	**Name**	***pI***		***MW***		***M***_***(G/TP)***_/*WT*_**(*G*/*TP*)**_	***M*_***(TP)***_/*WT*_(*TP*)_**	**# Unique peptides**		**# *N*-glyco sites**	***SP***	**LOC**	**References**
			***E***	***T***	***E***	***T***			***WT***	***M***				
49	C0PD06	Uncharacterized protein	5.77	7.26	87.29	56.24	0.45	0.88	7	5	2	0.14		
493	B6TX01	Glycoside hydrolase, family 28	5.68	6.16	85.53	49.74	0.52	1.02	3	3	10	0.38	M
														Tawde and Freimuth, 2012
	B7ZXJ2	Simillar to Zinc finger protein-like from Oryza sativa (Q5YLY5)		6.79		48.15			4	4	2	0.11		
454	C0HF77	Simillar to Subtilisin-like protease from Zea mays (B6U0R8)	8.11	8	65.86	82.63	0.56	0.99	7	10	3	0.95	S	Bykova et al., 2006
376	B4FNK1	Simillar to Proteasome subunit beta type from Zea mays (B6TGL3)	5.5	5.45	16.10	40.51	0.61	1.63	13	10	1	0.05		Overath et al., 2012
471	B4FRC8	Fruit protein PKIWI52	4.91	6.62	17.51	31.61	0.62	1.52	7	6	0	0.00	C	
433	B6T504	Proteasome subunit alpha type	4.85	4.72	17.38	25.95	0.64	1.73	8	9	0	0.05		Overath et al., 2012
495	B8A2E9	Simillar to 2-isopropylmalate synthase B from Zea mays (B6SWN1)	5.78	6.59	71.07	68.68	0.65	1.49	9	3	4	0.00	C	
	C4J6I7	Simillar to Phosphoribosylaminoimidazole carboxylase atpase-subunit, putative from Ricinus communis (B9S7H9)		6.59		68.71			5	7	2	0.00	C	
362	Q9ZP62	Glutathione transferase III(A)	6.47	5.96	16.45	23.92	0.67	1.99	3	3	1	0.31		Boušová et al., 2011
	B4FY73	Simillar to Rhicadhesin receptor from Zea mays (B6TKE1)		6.57		22.82			8	8	1	0.96	S	Gucciardo et al., 2007
451	B4FUT3	Germin-like protein subfamily 1 member 17	8.37	6.4	16.22	24.60	0.67	1.45	3	3	1	0.95	S	Gucciardo et al., 2007
461	C0HF77	Simillar to Subtilisin-like protease from Zea mays (B6U0R8)	7.92	8	65.56	82.63	0.67	1.08	6	14	3	0.95	S	Bykova et al., 2006
418	B4FVS8	Protein phosphatase 2C isoform epsilon	4.82	4.7	24.14	30.66	0.67	1.76	4	3	1	0.11		Alonso et al., 2004.
	B6TU39	Peroxidase 2		4.85		34.96			3	5	1	0.03		Spadiut et al., 2012
361	B4FUT3	Germin-like protein subfamily 1 member 17	6.94	6.4	16.51	24.60	0.67	2.26	3	3	1	0.95	S	Gucciardo et al., 2007
491	Q9LLB8	Exoglucanase	7.4	6.54	88.18	64.61	0.69	1.27	6	9	5	0.81	S	Grevesse et al., 2003
	C0PD60	Simillar to WD-repeat protein, putative from Ricinus communis (B9SSV2)		6.48		72.51			3	7	4	0.19		
	C0P3S2	Simillar to Protein TOC75, chloroplastic from Oryza sativa (Q84Q83)		7.64		91.63			18	12	5	0.00	C	
436	C0PFA1	Adenylosuccinate synthetase, chloroplastic	6.52	6.68	52.76	51.96	0.69	0.69	9	7	2	0.02	C	
	B6TBZ8	Alanine aminotransferase 2		6.23		53.03			6	7	1	0.09		Beránek et al., 2001
	B4FQK0	Simillar to Monodehydroascorbate reductase from Zantedeschia aethiopica (Q9SPM2)		8.65		53.77			3	13	0	0.00	M	Xue et al., 2012
	Q948J8	Uncleaved legumin-1		6.2		52.83			10	15	1	0.77	S	Arcalis et al., 2010
	B6TX10	6-phosphogluconate dehydrogenase, decarboxylating		6.3		54.17			4	3	1	0.07	C	
498	B4FUT3	Germin-like protein subfamily 1 member 17	7.75	6.4	16.36	24.60	0.70	2.45	3	4	1	0.95	S	Gucciardo et al., 2007
535	C0HHC3	Simillar to putative lipase from Hordeum vulgare (A1C0L3)	5.52	6.49	27.03	39.67	0.70	2.02	4	6	1	0.97	S	Romdhan et al., 2012
449	B4FW90	ER luminal binding protein	5.08	5.1	85.53	73.08	0.71	3.67	16	7	1	0.90	S	Denecke et al., 1991
	C4J410	Heat shock protein1		5.07		70.88			16	21	4	0.13		Koles et al., 2007
	B7ZZ42	Simillar to DnaK-type molecular chaperone hsp70-rice from Oryza sativa (Q53NM9)		5.1		71.16			9	9	5	0.07		Porras et al., 2005
	B6SZ69	Heat shock cognate 70 kDa protein 2		5.05		71.14			4	3	4	0.07		Koles et al., 2007.
	C0PF13	Simillar to Alpha-N-arabinofuranosidase A from Zea mays (B6T9B9)		5.2		74.85			6	10	3	0.02		Koseki et al., 2006
480	B6U0S1	Elongation factor 2	6.39	6	159.25	93.92	0.71	1.12	13	4	3	0.09		Solórzano et al., 2012
	C0HER4	Simillar to Putative aconitate hydratase 1 from Sorghum bicolor (Q1KSB0)		5.76		74.31			11	15	3	0.10		
641	Q948J8	Uncleaved legumin-1	6.66	6.2	52.33	52.83	0.71	0.88	15	10	1	0.77	S	Arcalis et al., 2010
371	B6UGQ9	Rhicadhesin receptor	6.47	6.57	16.27	22.83	0.72	2.01	5	9	1	0.97	S	Gucciardo et al., 2007
440	B4FW90	ER luminal binding protein	7.08	5.1	42.29	73.08	0.74	1.06	3	3	1	0.90	S	Denecke et al., 1991
	B4FUE0	GTP-binding protein PTD004		6.3		44.24			3	8	2	0.07		
	P93629	Alcohol dehydrogenase class-3		6.37		40.77			3	4	1	0.15		
	B6TYM9	Vignain		6.47		40.90			9	8	1	0.99	S	Müntz, 1996
357	B6UGQ9	Rhicadhesin receptor	6.47	6.57	16.57	22.83	0.74	1.83	6	7	1	0.97	S	Gucciardo et al., 2007
425	B4G0N0	Glucose-6-phosphate isomerase	7.73	6.96	63.82	62.24	0.75	1.16	15	15	4	0.15		
	C0HF77	Simillar to Subtilisin-like protease from Zea mays (B6U0R8)		8		82.63			14	14	3	0.95	S	Bykova et al., 2006
272	B4FNM4	60S acidic ribosomal protein P0	5.27	5.19	26.68	34.49	0.77	1.58	12	11	1	0.52		Zhou et al., 2007
	B6TP93	Fructokinase-2		5.34		35.53			18	22	0	0.41		
478	B4FW31	Protein phosphatase 2C isoform epsilon	4.9	4.76	23.85	31.21	0.78	1.39	8	12	3	0.13		Lu, 2008
319	B6TR84	Glycine-rich RNA-binding protein 7	4.73	4.87	19.39	25.11	0.78	1.11	6	8	2	0.07	M	
187	C0P558	Simillar to Heterogeneous nuclear ribonucleoprotein A3-like protein 2 from Zea mays (B6TV58)	6.7	8.46	44.91	44.75	0.79	1.50	9	7	0	0.06		
	B4FLA2	Simillar to Chorismate synthase from Sorghum bicolor (C5WQV1)		7.64		47.07			4	4	2	0.00	C	
	B4FLJ3	Isocitrate dehydrogenase [NADP]		6.57		46.10			4	4	3	0.15		
435	B7ZYR3	Uncharacterized protein	6.92	6.3	89.35	67.25	0.79	2.03	5	5	2	0.01	C	
	Q9LLB8	Exoglucanase		6.54		64.61			4	3	5	0.81	S	Grevesse et al., 2003
	C4J692	Simillar to Protein TOC75, chloroplastic from Oryza sativa (Q84Q83)		6.91		73.86			7	5	4	0.10	M	
649	P12863	Triosephosphate isomerase, cytosolic	5.46	5.52	16.83	26.89	0.79	1.06	5	11	2	0.05		Love and Hanover, 2005
	B6UB73	APx1-Cytosolic Ascorbate Peroxidase		5.65		27.39			4	9	0	0.04		
626	B6SMQ5	Triosephosphate isomerase	5.28	5.5	17.36	27.34	0.80	0.94	7	11	2	0.09		Love and Hanover, 2005
247	B6TS23	Protein Z	5.43	5.6	28.48	42.16	0.80	0.93	3	4	1	0.01	C	Spiro, 2002
	Q06509	Caffeic acid 3-0-methyltransferase		5.48		39.57			13	10	1	0.30		
	B4FTN5	Simillar to Protein MYG1, putative from Ricinus communis (B9RYS8)		6.02		42.70			5	4	3	0.01	M	
513	B8A3D0	Simillar to Chloroplast heat shock protein 70 from Pennisetum americanum (A4ZYQ0)	4.96	4.84	87.29	66.47	1.19	1.08	16	10	6	0.05		Koles et al., 2007
	B6UFB3	Stromal 70 kDa heat shock-related protein		5.08		74.67			9	29	6	0.01	C	Koles et al., 2007
590	B7ZX15	Simillar to Putative diphosphate-fructose-6-phosphate 1-phosphotransferase from Oryza sativa (Q6Z522)	7.73	8.23	54.66	58.19	1.19	0.91	6	3	3	0.03	M	
	B4FBF4	Serine hydroxymethyltransferase		6.84		51.60			14	8	1	0.13		
	B6T7Q7	Serine hydroxymethyltransferase		8.6		56.53			6	9	2	0.02	M	
403	C0P2V1	Simillar to Leucine aminopeptidase 2, chloroplastic from Oryza sativa (Q6K669)	5.22	5.62	61.20	28.37	1.48	0.76	5	4	3	0.13		Matsushita-Morita et al., 2011
	B4F8W6	Simillar to UTP–glucose-1-phosphate uridylyltransferase from Zea mays (B6T4R3)		5.3		52.09			4	5	2	0.16		Eimert et al., 1996
	B4FAD9	Simillar to UTP–glucose-1-phosphate uridylyltransferase from Zea mays (B6T4R3)		5.23		52.18			20	16	2	0.13		Eimert et al., 1996
547	B9TSW1	Glutamine synthetase	5.12	5.25	35.39	39.31	2.35	1.01	4	6	3	0.15		Shin and Park, 2004
	B6TMW7	Transaminase/ transferase, transferring nitrogenous groups		5.07		43.53			4	6	0	0.12		
	B6TS21	Succinyl-CoA ligase beta-chain		5.99		45.19			8	4	0	0.01	M	

pI-isoelectric point, MW-molecular weight, E-experimental observation, T-reported value, M_(*G*/*TP*)_/*WT*_(*G*/*TP*)_-normalized glycosylation ratio, *M*_(*TP*)_/*WT*_(*TP*)_–total protein fold change, #Unique peptides-number of unique peptides identified in MS, #N-glyco sites-number of predicted N-X-S or N-X-T glycosilation motifs, X is any amino acid except proline (http://www.hiv.lanl.gov/content/sequence/GLYCOSITE/glycosite.html), SP-Signal peptide prediction (http://www.cbs.dtu.dk/services/TargetP/), M-mitochondrion, C-chloroplast, S-secretory pathway.

**Figure 2:**
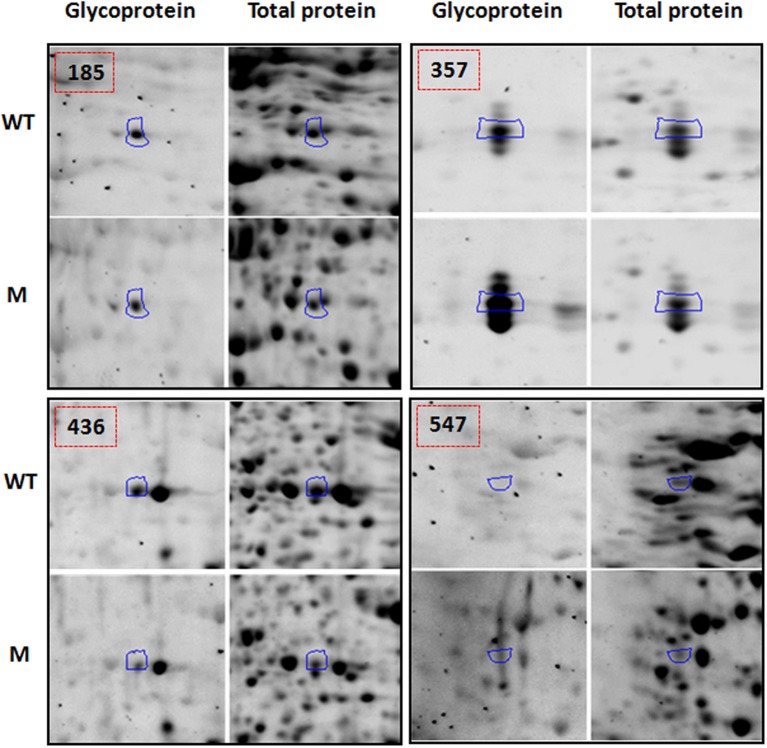
**Examples of protein spots from BETL total protein extracts [spot 185 [*M*_(*G*/*TP*)_/*WT*_(*G*/*TP*)_ = 0.97], spot 357 [*M*_(*G*/*TP*)_/*WT*_(*G*/*TP*)_ = 0.74], spot 436 [*M*_(*G*/*TP*)_/*WT*_(*G*/*TP*)_ = 0.69], and spot 547 [*M*_(*G*/*TP*)_/*WT*_(*G*/*TP*)_ = 2.35] showing quantitative differences in glycosylation (G) and total protein (TP) between WT and mutant after glycoprotein and Sypro Ruby staining**. The spots that showed a 20% increase/decrease (357, 436, and 547) in the glycosylation ratio were identified by mass spectrometry and labeled with the same numbers as in Table 1. Spots that did not showed significant changes in glycosylation ratios (185) are in Supplemental Table 1.

Figure 2 shows four representative spots with dynamic changes in normalized glycosylation levels (G/TP). The majority of proteins have decreased glycosylation ratio that is calculated by *M*_(*G*/*TP*)_/*WT*_(*G*/*TP*)_; as demonstrated by spots 357 and 436 in Figure 2 with ratios of 0.69 and 0.71, respectively. Only four spots showed 20% increased glycosylation in the mutant compared to the wild type. The spot 547 in Figure 2 is one of those proteins with glycosylation levels in the mutant being more than two-fold of those in the wild type. This indicates the relationship between glycosylation and protein abundance is complex. Figure 2 also includes an example of a protein, spot 185, with the glycosylation ratio of 0.97 that represented no significant change in glycosylation (Supplemental Table 1) in the two genotypes.

The 45 spots from the wild type and the mutant were excised and subjected to trypsin digestion and LC-MS/MS analysis. After database search using Mascot, the data were filtered in Scaffold software, and protein identification was considered valid if it had an overall 99% confidence and at least three unique peptides with 95% confidence. A total of 35 spots showed 69 comparable protein identifications between the WT and the M (Table 1). Some spots such as 272, 436, and 449 yielded multiple protein identifications due to the overlapping nature of proteins with similar molecular weights and isoelectric points (pIs). Several proteins were also reported by Zhu et al. (2006) in a similar proteomic study of root elongation zone in maize. Conversely, the same proteins were identified from multiple spots, e.g., spots 425, 454, and 461 as subtilisin-like protease, spots 357, 362, and 371 as Rhicadhesin receptor, and spots 440 and 449 as ER luminal binding protein. Differential post-translational modifications may account for these isoforms (Satoh et al., 2002). Alternative splicing is known to create multiple RNAs from a single gene that increase the proteome diversity. In maize, 19.2% of the total expressed genes are reported to be alternatively spliced (Barbazuk et al., 2008); thus various protein isoforms encoded by the same gene or its homologs may have alternative sizes and/or pIs.

Although nearly all the identified proteins (Table 1) exhibited close correlations between experimental and reported pIs, some did show discrepancy between the experimental molecular weights and theoretical molecular weights (e.g., spots 376, 454, and 493). These variations can be attributable to several factors, such as the lack of full length cDNA sequences or misannotation for these proteins; some of the cDNA sequences may have different splicing or degraded products, and post-translational modifications like glycosylation, which can also alter the molecular weights. Out of the 69 proteins presented in Table 1, 39 have been reported to be glycosylated.

## Functional classification

**Figure 3:**
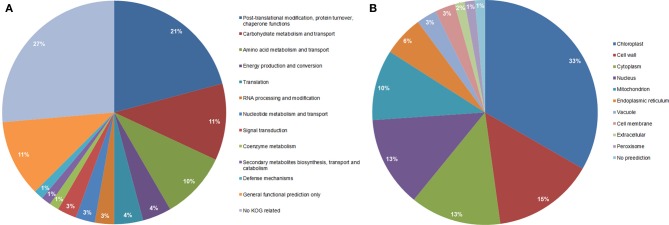
**Functional classification (A), and subcellular localization prediction (B), of the 69 proteins identified by LC-MS/MS in total protein extracts**.

Functional classification of the identified proteins (Table 1) was performed according to the clusters of orthologous groups for eukaryotic complete genomes (KOG, Tatusov et al., 2003). Thirteen different categories were found: (I) Post-translational modification, protein turnover, chaperone functions; (II) carbohydrate metabolism and transport; (III) Amino acid metabolism and transport; (IV) Energy production and conversion; (V) Translation; (VI) RNA processing and modification; (VII) Nucleotide metabolism and transport; (VIII) Signal transduction; (IX) Coenzyme metabolism; (X) Secondary metabolites biosynthesis, transport and catabolism; (XI) Defense mechanisms; (XII) General functional prediction only; (XIII) No KOG related; as shown in Figure 3A.

Category I (Post-translational modification, protein turnover, chaperone functions) was the most represented category that included 15 proteins such as ER luminal binding protein (spots 440 and 449, Table 1), glutathione transferase (spot 362), heat shock proteins (spots 449 and 513), proteasome subunits (spots 376 and 433), chaperone proteins (spot 449), vignain (spot 440), subtilisin like protease (spots 454, 461, and 425), and WD repeat (spot 491). Interestingly, all proteins represented in the 10 spots are glycosylated as reported previously in other organisms (Table 1, the last column for the references); however, all showed reduced normalized glycosylation ratio except for the heat shock protein 70 (spot 513) with a ratio of 1.19. These spots showed a total protein ratio, *M*_(*TP*)_/*WT*_(*TP*)_, of 1.0 or slightly higher (Table 1), suggesting that the reduced glycosylation in the mutant was not associated with the reduced abundance of these proteins.

Protein glycosylation is accomplished when the lipid (dolichol)-linked oligosaccharide transfers the carbohydrate-containing structure (Glc_3_Man_9_GLcNAc_2_) to nascent peptides in the ER (Howell, 2013).Several studies have shown that the glucose residues on the lipid linked oligosaccharide facilitate the *in vitro* transfer of the oligosaccharide to the protein although it is not an absolute requirement (Kornfeld and Kornfeld, 1985). Cells starved of glucose or in energy deprivation state fail to produce Glc_3_Man_9_GlcNAc_2_-P-P-Dol; instead, they accumulate Man_9_GlcNAc_2_ lipid linked species that were associated with the decrease in protein glycosylation in thyroid slices (Spiro et al., 1983). The accumulation of defective saccharide-lipid linked carrier upon different stresses varies according to cell type. In the BETL region of the *mn*1 mutant kernels, the low glucose content may lead to defective or low levels of the dolichol-linked oligosaccharide causing a reduction of the normal glycosylation process associated as a general trend of low glycosylation ratios when compared with the WT.

Table 1 depicts proteins with a high total protein ratio when comparing to the wild type, including heat shock proteins (spots 449 and 513), glutathione transferase III (spot 362) and proteasome (spots 376 and 433). Proteomic studies in developing seeds of *Brassica campestri L* reported that 23.4% of the total proteins are involved in protein processing and degradation (Li et al., 2012b).

One of the main protein folding machineries in protein processing is the *N*-glycan dependent folding pathway (Howell, 2013). After the Glc_3_Man_9_GlcNAc_2_ core is transferred to the nascent polypeptide by the oligosaccharide transferase (OST), the Glc α-1, 2, and α-1,3 are removed by the glucosidase I and II, then calreticulin/calnexin along with protein disulfide isomerase (PDI) help in the proper folding of the protein. Folded proteins are released by glucosidase which cleaves off the last glucose α-1,3 and are finally secreted. Proteins that are not properly folded are sensed by UDP-glucose:glycoprotein glucosyltransferase, and they can be reglucosylated to reenter the calreticulin/calnexin mediated folding cycle; where N-glycosylation plays an important role in the endoplasmic reticulum quality control (ERQC). We hypothesized that ER stress was induced in the BETL region due to the observed low protein N-glycosylation, and since *N*-glycans are recognized along several steps in the folding process of proteins, low N-glycosylation may lead to accumulation of unfolded/misfolded proteins that triggers the UPR response to mitigate the ER stress. These observations are consistent with Howell (2013) that UPR in plants is associated with upregulation of genes involved in protein folding and endoplasmic reticulum associated degradation (ERAD).

We observed four Hsp1 and Hsp70 proteins (spots 449 and 513); some with a high protein ratio in the *mn*1 mutant kernels when compared with the WT; these proteins are reported to be involved in protein targeting to the mitochondria and chloroplasts (Kriechbaumer et al., 2012), along with ER luminal binding proteins (spots 440 and 449), proposed to be involved in protein body assembly within the ER (Li et al., 2012b), supporting the hypothesis of ER stress and UPR process as a response of the defective glycosylation process occurring in the BETL region.

In addition to UPR response, the degradation of proteins via the ubiquitination-mediated pathway is an important mechanism in plant growth and development, and is responsible for the degradation of abnormal peptides and short-lived cellular regulators; controlling many processes that allow rapid response to intracellular signals (Sadanandom et al., 2012).This pathway is also related the ERAD by sensing glycoproteins that fail to fold into a native state. The alpha mannosidase interfere the reglucosylation cycles and removes the α-1,6 linked mannose from the core oligosaccharide linked to a glycoprotein. The mannose trimmed glycoprotein is recognized by a series of luminal proteins such as Lectin OS9 and HRD3 and then exported to the cytosol to be degraded via ubiquitinilation mediated pathway (Howell, 2013). The observation of proteasome subunit alpha and beta (spots 433 and 376 respectively) in a high total protein ratio in the *mn*1 mutant compared to the WT kernels suggest that *mn*1 kernels are undergoing ER stress due to low concentration of glucose in the BETL region.

A correlation between ER stress and programed cell death (PCD) is also described in plants (Zuppini et al., 2004). PCD occurs when internal contents of a cell are engulfed in the vacuole, leading to the tonoplast rupture and release of vacuolar hydrolytic enzymes. The tonoplast rupture is mediated by vacuolar processing enzymes that have caspase–like activity (Howell, 2013). Along with the observed proteins related to ER stress, UPR, and ERAD processes, we found that the subtilisin-like protease (spots 425, 454, and 461) also have a reported caspase-like activity (Vartapetian et al., 2011). This protein showed a low normalized glycosylation level and no change in the total protein ratio in the *mn*1 mutant compared to the WT, suggesting that ER stress might regulate PCD through under-glycosylation in the *mn*1 mutant BETL region. Overall, the reduced glycosylation levels of many of the above proteins may also affect diverse metabolic and cellular functions in developing seeds.

Carbohydrate catabolism and transport, the second most represented group in the functional classification (Figure 3A), is composed of eight proteins in the glycolysis pathway, pentose pathway, fructose and mannose metabolism and starch synthesis (spots 272, 403 with two proteins, 425, 436, 590, 626, and 649). The enzymes that participate in glycolysis pathway showed low ratios of glycosylation in the *mn*1 mutant when compared to WT and no change in the total protein ratio (Table 1). Several glycolytic enzymes among other protein/enzymes are reported to be modified by *O*-GlcNAc in various systems (Love and Hanover, 2005). The enzymes that participate in glycolysis are of special interest, due to the greatly reduced levels of hexoses resulting from the INCW2-deficiency in the BELT region of the mutant.

One of the proteins involved in carbohydrate catabolism is glucose-6-phosphate-isomerase (spot 425). It catalyzes the conversion of glucose-6-phosphate to fructose 6-phosphate, an important step in the hexoamine biosynthetic pathway. We observed that the glycosylation ratio of glucose-6-phosphate-isomerase was reduced (≈0.76) and the total protein ratio was nearly the same (≈1.16), suggesting that its enzymatic function and activity may be modulated through glycosylation. Cells can sense the level of a product, UDP-GlcNAc, in the hexoamine biosynthetic pathway as an indication of the level of glucose, the precursor that is used in the same pathway (Love and Hanover, 2005). Because glucose-6-phosphate-isomerase is an essential enzyme in the hexoamine biosynthetic pathway, we hypothesize that it plays an important role in maintaining the homeostasis in the cells in terms of nutrient levels in the *mn*1 kernel. Further studies are needed in the characterization of this particular protein and its role in regulatory pathways.

Triosephosphate isomerase (spot 649) that catalyzes the interconversion of glyceraldehyde-3-phosphate to dihydroxyacetone phosphate was found in cytosolic and plastid isoforms with the same trend in glycosylation and total protein abundance as the glycolytic enzymes, in which glycosylation level goes down while the total protein level doesn't change significantly. For fructose and mannose metabolism, fructokinase-2 (spot 272) was found with a low glycosylation ratio but a high ratio in total protein (≈1.5); while diphosphate-fructose-6-phosphate 1-phosphotransferas (spot 590) was found with a high ratio of glycosylation (≈1.2) and no significant change in the total protein ratio. The 6-phosphogluconate dehydrogenase (spot 436), a decarboxylating enzyme, participates in the pentose phosphate pathway (PPP) and was found with low ratios for both glycosylation and total protein. Significantly, Spielbauer et al. (2013), reported a subset of chloroplast-localized PPP enzymes to be not only present in endosperm amyloplasts but are also critical for starch and oil biosynthesis in developing seeds. The UTP-glucose-1-phosphate uridylyltransferase (spot 403) is responsible for synthesis and pyrophosphorolysis of UDP-glucose, a key precursor of carbohydrate formation (including sucrose, cellulose, starch, glycogen and β-glucan biosynthesis). This protein showed one of the highest ratios in glycosylation but had a reduced ratio in total protein level. UGPase is highly abundant in cell wall membrane in barley and it is speculated to have a role in providing UDP-glucose for biosynthesis of cell wall components, including β-glucans and cellulose (Eimert et al., 1996).

Amino acid metabolism and transport was the third most important category in functional classification. There were six proteins involved in amino acid metabolism; 2-isopropylmalate synthase, alanine aminotransferase, chorismate synthase, serine hydroxymethyltransferase, transaminase and glutamine synthetase (spots 495, 436, 187, 590, and 547 for last two proteins). The first three enzymes showed a low glycosylation ratio but 2-isopropylmalate synthase and chorismate synthase had a high protein abundance ratio (Table 1). The last three enzymes had high glycosylation ratios but did not change in protein levels, which suggests that glycosylation may play a role in the regulation of these enzymes. In rice seed development proteomics studies, Deng et al. (2013) observed a correlation between the accumulation of proteins involved in protein synthesis and turnover, protein folding and amino acid metabolism, in early stages of rice seed development, indicating an active turnover of proteins during early development stages; as observed in our work.

### Subcellular location of the identified proteins

When observing the subcellular location prediction of the common proteins between the wild type and the mutant (Figure 3B), the chloroplast was the most represented with a 33% contribution of the total proteins identified, followed by the cell wall proteins (15%) and cytoplasm (13%). The chloroplast protein group consists of a very wide spectrum of different proteins that are involved in energy production and conversion, amino acid metabolism and transport, nucleotide metabolism and transport, carbohydrate metabolism and transport, and post-translational modification-protein turnover-chaperone functions.

All cell wall associated proteins showed low glycosylation level in the *mn*1 mutant compare to the WT, as expected. The cell wall group was composed of 10 proteins; three subtilisin-like proteases that have post-translational modification, protein turnover, and chaperone functions (spots 425, 454, and 461) did not show any significant change in their total protein ratio. The other seven proteins did not belong to any KOG related group and were germinin-like proteins (GLPs), rhicadhesin receptor, and alpha-*N*-arabinofuranosidase A (spots 357, 361, 362, 371, 449, 451, and 498), It has been reported that about 40% of all known GLPs are cell wall proteins; and all GLPs contain N-terminal secretory sequences that relate them strongly to the cell wall/extracellular matrix targeting (Breen and Bellgard, 2010). GLPs have been studied as defense molecules in different plant species, conditions and diseases due to their high resistance to protease, heat, SDS and extreme pH. Several reports identified GLPs in diverse environmental conditions, such as salt, aluminum, drought stresses, fungal pathogen attacks, bacteria and virus infections (Breen and Bellgard, 2010). A special class of GLPs that is almost exclusively found in cereals is characterized by an oxalate oxidase (OXO) activity; meanwhile, a barley GLP reported by Bernier and Berna (2001) presents a different ADP glucose enzyme activity that leads to the hypothesis of its involvement in the control of metabolic flow toward starch, cell wall polysaccharides, glycoproteins and glycolipids in plants (Rodríguez-López et al., 2001). In addition, Gucciardo et al. (2007) isolated a pea GLP that showed similarity in its N-terminus with a rhicadhesin recepetor. The GLP mRNA was expressed in non-conventional locations for rhicadhesin receptors such as in nodules and the expanding cells adjacent to the nodule meristem and in the nodule epidermis. This protein is demonstrated to have a superoxide dismutase activity and show resistance to high temperature among other stresses (Breen and Bellgard, 2010). All GLP proteins showed an increased protein ratio in the *mn*1 mutant kernel, suggesting an upregulation due to the stress caused by the glucose-deficiency in BETL region.

Eight proteins of cytosolic location (spots 362, 403, 418, 425, 440, 480, 491, and 547) are described in Table 1 and Figure 3B were found in the experiment. These proteins have a variety of functions such as amino acid metabolism and transport; carbohydrate metabolism and transport; post-translational modification, protein turnover, chaperone functions; secondary metabolites biosynthesis; transport and catabolism; and translation. Glutathione transferase III(A), peroxidase 2, and a like-WD-repeat protein (spots 362, 418, and 491; respectively) showed low normalized glycosylation level but an increase in the total protein ratio. These proteins are related to oxidative stress. In a previous study (Silva-Sanchez et al., 2013), proteins related to gluthatione metabolism were up-regulated, in the *mn*1 kernels, suggesting a potential redox regulation by glutathione. Elongation factor 2, alcohol dehydrogenase class-3 and glucose-6-phosphate isomerase (spots 425, 440, and 480; respectively) showed a low normalized glycosylation level and nearly no change in their total protein ratio. These observations suggest that their regulation may be mediated through their glycosylation. A protein similar to UTP-glucose-1-phosphate uridylyltransferase (UGPUT) from *Zea mays* (B6T4R3, spot 403) showed a high normalized glycosylation ratio (≈1.48) but a low total protein ratio (≈0.76). This protein plays an important role in the biosynthesis of cell wall polysaccharides as well as in the synthesis of the carbohydrate moiety for glycolipid and glycoproteins (Kleczkowski et al., 2004). Glutamine synthetase (GS) (spot 547) showed the highest level of normalized glycosylation (≈2.35) but no change in total protein ratio (≈1.01). Cytosolic GS participate in seed development by assimilating ammonium in sink tissues probably through the asparagine catabolism as a possible source of ammonium (Bernard and Habash, 2009). The high glycosylation levels of UGPUT and GS suggest a complex mode of post-translational regulation in the plant. It is interesting to add that glycosylation of cytosolic proteins has been reported previously. Haltiwanger et al. (1992) described that several cytoplasmic and nuclear proteins undergo *O*-glycosylation, which is highly dynamic in nature and is known to response to various stimuli in the media, in a similar way to phosphorylation responses. These processes are thus far well known in yeast or animal models (Spiro, 2002; Love and Hanover, 2005) although there is some research done in plants (Thornton et al., 1999; Love and Hanover, 2005 and references there in).

### *N*-glycosylation prediction and secretory pathway

For all of the proteins present in Table 1, we predicted the number of probable *N*-glycosylation sites based on the N-X-S, N-X-T, or NX[ST]Z motifs, where X can be any amino acid residues except proline using the *N*-glycosite tool (Zhang et al., 2004). A total of 48 proteins showed at least one predicted *N*-glycosylation site in their sequence. Those proteins that did not show any predicted *N*-glycosylation sites potentially have other types of unusual or rare glycosylation or are not studied/reported as glycoproteins yet. Although some proteins that showed at least one predicted *N*-glycosylation site, they may have been reported with other types of glycosylation. For example, the proteasome alpha subunit (spot 433) has been reported to be O-glycosylated in mouse (Overath et al., 2012); elongation factor 2 (spot 480) is an O-glycosylated protein in human cervical cancer cells (Solórzano et al., 2012), triosaphosphate isomerase (Spots 626 and 649) (Love and Hanover, 2005); and protein Z showed O-glycosylation in human plasma (Spiro, 2002). O-glycosylation occurs in a great variety of proteins that are involved in key nuclear and cytoplasmic activities. Similar to phosphorylation, *O*-GlcNAc modification occurs on Ser or Thr residues. *O*-GlcNAc is interchangeable with phosphorylation. These two modifications have similar roles in biological functions as well. *O*-GlcNAc modification is associated to a variety of regulatory activities such as carbohydrate metabolism, signaling, transcription and translation, and stress response (Love and Hanover, 2005).

In order to investigate whether the proteins are related to the secretory pathway and have the potential of being glycosylated or not, the identified proteins from the 45 spots in Table 1 were subjected to signal peptide prediction using the TargetP tool (Emanuelsson et al., 2000). According to the prediction, 17 proteins showed the highest scores for SP and have signal peptides. Examples of these proteins included rhicadhesin receptor (spots 357, 362, and 371), ER luminal binding protein (spots 440 and 449), GLP's (spots 361, 451, and 498), subtilisin-like protease (spots 425, 454, and 461). Many of these proteins are cell wall proteins or membrane proteins and are known to be glycosylated (Denecke et al., 1991; Bykova et al., 2006; Koseki et al., 2006; Gucciardo et al., 2007). For proteins with relative low scores for signal peptide prediction, eight proteins were assigned to mitochondria while 10 were located in the chloroplasts. The majority of these proteins have not been reported as glycoproteins. For the proteins that were not assigned neither to mitochondria or chloroplast; the low score on the SP prediction could indicate that these proteins have no classical secretion signal (Radhamony and Theg, 2006), and may follow so-called unconventional secretion based on alternative models of secretion independent of ER and Golgi route (Zhang and Schekman, 2013).

## Conclusions

The study of glycosylated proteins in the total protein extracts of BETL from wild type and the *mn*1 mutant revealed the deficiency of the mutant in the glycosylation process. The identified proteins that participate actively in post-translational modifications, protein turnover and chaperone functions may indicate the ER stress and UPR in the mutant due to the defective glycosylation processes. Carbohydrate metabolism related proteins showed in general low glycosylation ratios, suggesting that their enzymatic functions and activities may be regulated by the glycosylation levels through the hexosamine pathway. Low glycosylation ratios were also found in cell wall proteins of the mutant, which represent most of the plant defense proteins and structural protein-crosslink. More studies are needed to fully understand the mechanism underlying protein glycosylation, sugar sensing and plant survival.

### Conflict of interest statement

The authors declare that the research was conducted in the absence of any commercial or financial relationships that could be construed as a potential conflict of interest.

## Supplementary material

Supplemental Figure 1 **Overview of glycoprotein stain (A,B) vs. total protein stain (C,D) of wild type (A,C) and mutant (B,D)**.

Supplemental Table 1 **Selection of spots to be analyzed by mass spectrometry.** Spots in bold showed a significant variation of 20% and were chosen for mass spectrometry. GLY/G and TP stand for glycoproteins and total proteins.

## References

[B1] Alonso A. Sasin J. Bottini N. Friedberg I. Friedberg I. Osterman A. (2004). Protein tyrosine phosphatases in the human genome. Cell 117, 699–711 10.1016/j.cell.2004.05.018 15186772

[B2] Arcalis E. Stadlmann J. Marcel S. Drakakaki G. Winter V. Rodriguez J. (2010). The changing fate of a secretory glycoprotein in developing maize endosperm. Plant Physiol. 153, 693–702 10.1104/pp.109.152363 20388665 PMC2879800

[B3] Barbazuk W. B. Fu Y. McGinnis K. M. (2008). Genome-wide analysis of alternative splicing in plants: opportunities and challenges. Genome Res. 18, 1381–1392 10.1101/gr.053678.106 18669480

[B4] Beránek M. Drsata J. Palicka V. (2001). Inhibitory effect of glycation on catalytic activity of alanine aminotransferase. Mol. Cell. Biochem. 218, 35–39 10.1023/A:1007280913732 11330835

[B5] Bernard S. M. Habash D. Z. (2009). The importance of cytosolic glutamine synthetase in nitrogen assimilation and recycling. New Phytol. 182, 608–620 10.1111/j.1469-8137.2009.02823.x 19422547

[B6] Bernier F. Berna A. (2001). Germins and germin-like proteins: plant do-all proteins. But what do they do exactly? Plant Physiol. Biochem. 39, 545–554 10.1016/S0981-9428(01)01285-2

[B7] Boušová I. Prùchová Z. Trnková L. Dršata J. (2011). Comparison of glycation of glutathione S-transferase by methylglyoxal, glucose or fructose. Mol. Cell. Biochem. 357, 323–330 10.1007/s11010-011-0903-5 21625951

[B8] Breen J. Bellgard M. (2010). Germin-like proteins (GLPs) in cereal genomes: gene clustering and dynamic roles in plant defence. Funct. Integr. Genomics. 10, 463–476 10.1007/s10142-010-0184-1 20683632

[B9] Bykova N. V. Rampitsch C. Krokhin O. Standing K. G. Ens W. (2006). Determination and characterization of site-specific *N*-glycosylation using MALDI-Qq-TOF tandem mass spectrometry: case study with a plant protease. Anal. Chem. 78, 1093–1103 10.1021/ac0512711 16478099

[B10] Cheng W. H. Chourey P. S. (1999). Genetic evidence that invertase-mediated release of hexoses is critical for appropriate carbon partitioning and normal seed development in maize. Theor. Appl. Genet. 98, 485–495 10.1007/s001220051096

[B11] Cheng W. H. Taliercio E. W. Chourey P. S. (1996). The Miniature1 seed locus of maize encodes a cell wall invertase required for normal development of endosperm and maternal cells in the pedicel. Plant Cell 8, 971–983 12239408 10.1105/tpc.8.6.971 PMC161152

[B12] Chou K. C. Shen H. B. (2010). Plant-mPLoc: a top-down strategy to augment the power for predicting plant protein subcellular localization. PLoS ONE 5:e11335 10.1371/journal.pone.0011335 20596258 PMC2893129

[B13] Chourey P. S. Jain M. Li Q. B. Carlson S. J. (2006). Genetic control of cell wall invertase in developing endosperm of maize. Planta 223, 159–167 10.1007/s00425-005-0039-5 16025339

[B14] Chourey P. S. Li Q. B. Cevallos-Cevallos J. (2012). Pleiotropy and its dissection through a metabolic gene *Miniature1* (*Mn1*) that encodes a cell wall invertase in developing seeds. Plant Sci. 184, 45–53 10.1016/j.plantsci.2011.12.011 22284709

[B15] Davis R. W. Smith J. D. Cobb B. G. (1990). A light and electron-microscope investigation of the transfer cell region of maize caryopses. Can. J. Bot. 68, 471–479 10.1139/b90-063

[B16] Denecke J. Goldman M. H. Demolder J. Seurinck J. Botterman J. (1991). The tobacco luminal binding protein is encoded by a multigene family. Plant Cell 3, 1025–1035 1822990 10.1105/tpc.3.9.1025 PMC160068

[B17] Deng Z. Y. Gong C. Y. Wang T. (2013). Use of proteomics to understand seed development in rice. Proteomics 13, 1784–1800 10.1002/pmic.201200389 23483697

[B18] Eimert K. Villand P. Kilian A. Kleczkowski L. A. (1996). Cloning and characterization of several cDNAs for UDP-glucose pyrophosphorylase from barley (*Hordeum vulgare*) tissues. Gene 170, 227–232 10.1016/0378-1119(95)00873-X 8666250

[B19] Emanuelsson O. Nielsen H. Brunak S. von Heijne G. (2000). Predicting subcellular localization of proteins based on their N-terminal amino acid sequence. J. Mol. Biol. 300, 1005–1016 10.1006/jmbi.2000.3903 10891285

[B20] Grevesse C. Lepoivre P. Jijakli M. H. (2003). Characterization of the exoglucanase-encoding gene *PaEXG2* and study of its role in the biocontrol activity of *Pichia anomala* strain K. Phytopathology 93, 1145–1152 10.1094/PHYTO.2003.93.9.1145 18944099

[B21] Gucciardo S. Wisniewski J. P. Brewin N. J. Bornemann S. (2007). A germin-like protein with superoxide dismutase activity in pea nodules with high protein sequence identity to a putative rhicadhesin receptor. J. Exp. Bot. 58, 1161–1171 10.1093/jxb/erl282 17244628

[B22] Haltiwanger R. S. Kelly W. G. Roquemore E. P. Blomberg M. A. Dong L. Y. Kreppel L. (1992). Glycosylation of nuclear and cytoplasmic proteins is ubiquitous and dynamic. Biochem. Soc. Trans. 20, 264–269 1397609 10.1042/bst0200264

[B23] Howell S. H. (2013). Endoplasmic reticulum stress responses in plants. Annu. Rev. Plant Biol. 64, 477–499 10.1146/annurev-arplant-050312-120053 23330794

[B24] Hurkman W. J. Tanaka C. K. (1986). Solubilization of plant membrane proteins for analysis by two-dimensional gel electrophoresis. Plant Physiol. 81, 802–806 10.1104/pp.81.3.802 16664906 PMC1075430

[B25] Kang B. H. Xiong Y. Williams D. S. Pozueta-Romero D. Chourey P. S. (2009). Miniature1-encoded cell wall invertase is essential for assembly and function of wall-in-growth in the maize endosperm transfer cell. Plant Physiol. 151, 1366–1376 10.1104/pp.109.142331 19759348 PMC2773079

[B26] Kleczkowski L. A. Geisler M. Ciereszko I. Johansson H. (2004). UDP-glucose pyrophosphorylase. An old protein with new tricks. Plant Physiol. 134, 912–918 10.1104/pp.103.036053 15020755 PMC523891

[B27] Koles K. Lim J. M. Aoki K. Porterfield M. Tiemeyer M. Wells L. (2007). Identification of N-glycosylated proteins from the central nervous system of *Drosophila melanogaster*. Glycobiology 17, 1388–1403 10.1093/glycob/cwm097 17893096

[B28] Kornfeld R. Kornfeld S. (1985). Assembly of asparagine-linked oligosaccharides. Annu. Rev. Biochem. 54, 631–664 10.1146/annurev.bi.54.070185.003215 3896128

[B29] Koseki T. Miwa Y. Mese Y. Miyanaga A. Fushinobu S. Wakagi T. (2006). Mutational analysis of N-glycosylation recognition sites on the biochemical properties of *Aspergillus kawachii* alpha-L-arabinofuranosidase 54. Biochim. Biophys. Acta 1760, 1458–1464 10.1016/j.bbagen.2006.04.009 16784813

[B30] Kriechbaumer V. von Löffelholz O. Abell B. M. (2012). Chaperone receptors: guiding proteins to intracellular compartments. Protoplasma 249, 21–30 10.1007/s00709-011-0270-9 21461941

[B31] LeClere S. Schmelz E. A. Chourey P. S. (2008). Cell wall invertase-deficient miniature 1 kernels have altered phytohormone levels. Phytochemistry 69, 692–699 10.1016/j.phytochem.2007.09.011 17964617

[B32] LeClere S. Schmelz E. A. Chourey P. S. (2010). Sugar levels regulate tryptophan-dependent auxin biosynthesis in developing maize kernels. Plant Physiol. 153, 306–318 10.1104/pp.110.155226 PMC2862422 20237017

[B33] Li J. Ferraris J. D. Yu D. Singh T. Izumi Y. Wang G. (2012a). Proteomic analysis of high NaCl-induced changes in abundance of nuclear proteins. Physiol. Genomics 44, 1063–1071 10.1152/physiolgenomics.00068.2012 22991206 PMC3615577

[B34] Li W. Gao Y. Xu H. Zhang Y. Wang J. (2012b). A proteomic analysis of seed development in *Brassica campestri* L. PLoS ONE 7:e50290 10.1371/journal.pone.0050290 23189193 PMC3506616

[B35] Liu J. X. Howell S. H. (2010). Endoplasmic reticulum protein quality control and its relationship to environmental stress responses in plants. Plant Cell 22, 2930–2942 10.1105/tpc.110.078154 20876830 PMC2965551

[B36] Love D. C. Hanover J. A. (2005). The hexosamine signaling pathway: deciphering the “*O*-GlcNAc code.” Sci. STKE 2005, re13. 10.1126/stke.3122005re13 16317114

[B37] Lu G. (2008). Regulation of Local Signaling by Type 2c Protein Phosphatases. Dissertations, Academic, UCLA, Molecular Cellular, and Integrative Physiology, 66–67

[B38] Matsushita-Morita M. Tada S. Suzuki S. Hattori R. Marui J. Furukawa I. (2011). Overexpression and characterization of an extracellular leucine aminopeptidase from *Aspergillus oryzae*. Curr. Microbiol. 62, 557–564 10.1007/s00284-010-9744-9 20803144

[B39] Miller M. E. Chourey P. S. (1992). The maize invertase-deficient miniature-1 seed mutation is associated with aberrant pedicel and endosperm development. Plant Cell. 4, 297–305 12297647 10.1105/tpc.4.3.297 PMC160130

[B40] Müntz K. (1996). Proteases and proteolytic cleavage of storage proteins in developing and germinating dicotyledonous seeds. J. Exp. Botany. 47, 605–622 10.1093/jxb/47.5.605

[B41] Offler C. E. McCurdy D. W. Patrick J. W. Talbot M. J. (2003). Transfer cells: cells specialized for a special purpose. Annu. Rev. Plant Biol. 54, 431–454 10.1146/annurev.arplant.54.031902.134812 14502998

[B42] Overath T. Kuckelkorn U. Henklein P. Strehl B. Bonar D. Kloss A. (2012). Mapping of *O*-GlcNAc sites of 20 s proteasome subunits and Hsp90 by a novel biotin-cystamine tag. Mol. Cell Proteomics 11, 467–477 10.1074/mcp.M111.015966 22556278 PMC3412975

[B43] Porras F. Urrea F. Ortiz B. Martínez-Cairo S. Bouquelet S. Martínez G. (2005). Isolation of the receptor for the *Amaranthus leucocarpus* lectin from human T lymphocytes. Biochim. Biophys. Acta 1724, 155–162 10.1016/j.bbagen.2005.03.014 15866508

[B44] Radhamony R. N. Theg S. M. (2006). Evidence for an ER to Golgi to chloroplast protein transport pathway. Trends Cell. Biol. 16, 385–387 10.1016/j.tcb.2006.06.003 16815014

[B45] Rodríguez-López M. Baroja-Fernández E. Zandueta-Criado A. Moreno-Bruna B. Muñoz F. J. Akazawa T. (2001). Two isoforms of a nucleotide-sugar pyrophosphatase/phosphodiesterase from barley leaves (*Hordeum vulgare* l.) are distinct oligomers of hvglp1, a germin-like protein. FEBS Lett. 490, 44–48 11172808 10.1016/s0014-5793(01)02135-4

[B46] Romdhan I. B. Fendri A. Frikha F. Gargouri A. Belghith H. (2012). Purification, physico-chemical and kinetic properties of the deglycosylated *Talaromyces thermophilus* lipase. Int. J. Biol. Macromol. 51, 892–900 10.1016/j.ijbiomac.2012.06.034 22766036

[B47] Ruan Y. L. Jin Y. Yang Y. J. Li G. J. Boyer J. S. (2010). Sugar input, metabolism, and signaling mediated by invertase: roles in development, yield potential, and response to drought and heat. Mol Plant. 3, 942–955 10.1093/mp/ssq044 20729475

[B48] Ruiz-May E. Thannhauser T. W. Zhang S. Rose J. K. C. (2012). Analytical technologies for identification and characterization of the plant N-glycoproteome. Front. Plant Sci. 3:150 10.3389/fpls.2012.00150 22783270 PMC3389394

[B49] Sadanandom A. Bailey M. Ewan R. Lee J. Nelis S. (2012). The ubiquitin-proteasome system: central modifier of plant signalling. New Phytol. 196, 13–28 10.1111/j.1469-8137.2012.04266.x 22897362

[B50] Satoh K. Takeuchi M. Oda Y. Deguchi-Tawarada M. Sakamoto Y. Matsubara K. (2002). Identification of activity-regulated proteins in the postsynaptic density fraction. Genes Cells 7, 187–197 10.1046/j.1356-9597.2001.00505.x 11895482

[B51] Sheffield J. Taylor N. Fauquet C. Chen S. (2006). The cassava (*Manihot esculenta* Crantz) root proteome: protein identification and differential expression. Proteomics 6, 1588–1598 10.1002/pmic.200500503 16421938

[B52] Shin D. Park C. (2004). N-terminal extension of canine glutamine synthetase created by splicing alters its enzymatic property. J. Biol. Chem. 279, 1184–1190 10.1074/jbc.M309940200 14583610

[B53] Silva-Sanchez C. Chen S. Zhu N. Li Q.-B. Chourey P. S. (2013). Proteomic comparison of basal endosperm in maize *miniature*1 mutant and its wild-type *Mn*1. Front. Plant Sci. 4:211 10.3389/fpls.2013.00211 23805148 PMC3691554

[B54] Solórzano C. Angel Mayoral M. de los Angeles C. M. Berumen J. Guevara J. Raúl Chávez F. (2012). Overexpression of glycosylated proteins in cervical cancer recognized by the *Machaerocereus eruca* agglutinin. Folia Histochem. Cytobiol. 50, 398–406 10.5603/FHC.2012.0054 23042270

[B55] Spadiut O. Rossetti L. Dietzsch C. Herwig C. (2012). Purification of a recombinant plant peroxidase produced in *Pichia pastoris* by a simple 2-step strategy. Protein Expr. Purif. 86, 89–97 10.1016/j.pep.2012.09.008 23026679

[B56] Spielbauer G. Li L. Romisch-Margl L. Do T. Fouquet R. Fernie A. R. (2013). Chlorplast-localized 6-phosphogluconate dehydrogensae is critical for maize starch accumulation. J. Exp. Bot. 64, 2231–2242 10.1093/jxb/ert082 23530131 PMC3654415

[B57] Spiro R. G. (2002). Protein glycosylation: nature, distribution, enzymatic formation, and disease implications of glycopeptide bonds. Glycobiology 12, 43R–56R 10.1093/glycob/12.4.43R 12042244

[B58] Spiro R. G. Spiro M. J. Bhoyroo V. D. (1983). Studies on the regulation of the biosynthesis of glucose-containing oligosaccharide-lipids. Effect of energy deprivation. J. Biol. Chem. 258, 9469–9476 6874696

[B59] Tatusov R. L. Fedorova N. D. Jackson J. D. Jacobs A. R. Kiryutin B. Koonin E. V. (2003). The COG database: an updated version includes eukaryotes. BMC Bioinformatics 4:41 10.1186/1471-2105-4-41 12969510 PMC222959

[B60] Tawde M. D. Freimuth P. (2012). Toxic misfolding of Arabidopsis cellulases in the secretory pathway of *Pichia pastoris*. Protein Expr. Purif. 85, 211–217 10.1016/j.pep.2012.08.009 22929090

[B61] Thornton T. M. Swain S. M. Olszewski N. E. (1999). Gibberellin signal transduction presents ellipsisthe SPY who *O*-GlcNAc'd me. Trends Plant Sci. 4, 424–428 10.1016/S1360-1385(99)01485-5 10529823

[B62] Vartapetian A. B. Tuzhikov A. I. Chichkova N. V. Taliansky M. Wolpert T. J. (2011). A plant alternative to animal caspases: subtilisin-like proteases. Cell Death Differ. 18, 1289–1297 10.1038/cdd.2011.49 21546909 PMC3172098

[B63] Vaughn K. C. Talbot M. J. Offler C. E. McCurdy D. W. (2007). Wall ingrowths in epidermal transfer cells of *Vicia faba* cotyledons are modified primary walls marked by localized accumulations of arabinogalactan proteins. Plant Cell Physiol. 48, 159–168 10.1093/pcp/pcl047 17169921

[B64] Vitale A. Boston R. S. (2008). Endoplasmic reticulum quality control and the unfolded protein response: insights from plants. Traffic 9, 1581–1588 10.1111/j.1600-0854.2008.00780.x 18557840

[B65] Xue Y. L. Miyakawa T. Sawano Y. Tanokura M. (2012). Cloning of genes and enzymatic characterizations of novel dioscorin isoforms from *Dioscorea japonica*. Plant Sci. 183, 14–19 10.1016/j.plantsci.2011.10.021 22195572

[B66] Zhang M. Gaschen B. Blay W. Foley B. Haigwood N. Kuiken C. (2004). Tracking global patterns of N-linked glycosylation site variation in highly variable viral glycoproteins: HIV, SIV, and HCV envelopes and influenza hemagglutinin. Glycobiology 14, 1229–1246 10.1093/glycob/cwh106 15175256

[B67] Zhang M. Schekman R. (2013). Unconventional secretion, unconventional solutions. Scie 340, 559–561 10.1126/science.1234740 23641104

[B68] Zhou H. Liu Y. Chui J. Guo K. Shun Q. Lu W. (2007). Investigation on glycosylation patterns of proteins from human liver cancer cell lines based on the multiplexed proteomics technology. Arch. Biochem. Biophys. 459, 70–78 10.1016/j.abb.2006.10.027 17214954

[B69] Zhu J. Chen S. Alvarez S. Asirvatham V. S. Schachtman D. P. Wu Y. (2006). Cell wall proteome in the maize primary root elongation zone. I. Extraction and identification of water-soluble and lightly ionically bound proteins. Plant Physiol. 140, 311–325 10.1104/pp.105.070219 16377746 PMC1326053

[B70] Zhu M. Simons B. Zhu N. Oppenheimer D. G. Chen S. (2010). Analysis of abscisic acid responsive proteins in *Brassica napus* guard cells by multiplexed isobaric tagging. J. Proteomics 73, 790–805 10.1016/j.jprot.2009.11.002 19913118

[B71] Zuppini A. Navazio L. Mariani P. (2004). Endoplasmic reticulum stress-induced programmed cell death in soybean cells. J. Cell Sci. 117, 2591–2598 10.1242/jcs.01126 15159454

