# Supplementary material for: A comparative glycoproteome study of developing endosperm in the hexose-deficient miniature1 (mn1) seed mutant and its wild type Mn1 in maize
Source: Front Plant Sci. 2014 Feb 26;5:63. doi: 10.3389/fpls.2014.00063 (PMC3935489; doi:10.3389/fpls.2014.00063)
Supplement: Supplemental Figure 1 — Overview of glycoprotein stain (A,B) vs. total protein stain (C,D) of wild type (A,C) and mutant (B,D). [file DataSheet2.PDF]

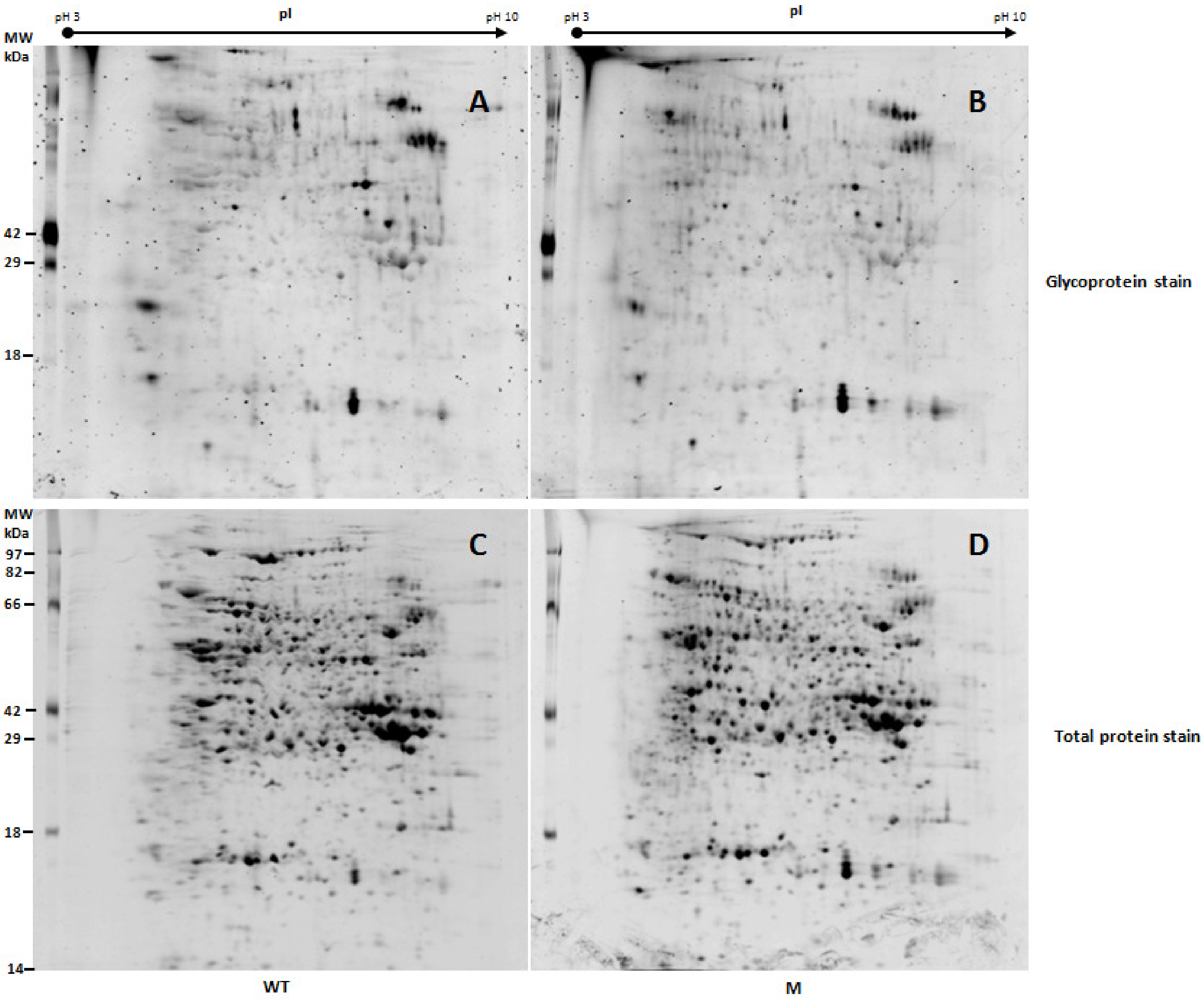

Supplemental Figure 1. Overview of glycoprotein stain (A, B) versus total protein stain (C, D) of wild type (A, C) and mutant (B, D).
